# Regulation of BCR-mediated Ca2+ mobilization by MIZ1-TMBIM4 safeguards IgG1+ GC B cell-positive selection

**DOI:** 10.1126/sciimmunol.adk0092

**Published:** 2024-04-05

**Authors:** Lingling Zhang, Amparo Toboso-Navasa, Arief Gunawan, Abdouramane Camara, Rinako Nakagawa, Finsterbusch Katja, Probir Chakravarty, Rebecca Newman, Yang Zhang, Martin Eilers, Andreas Wack, Pavel Tolar, Kai-Michael Toellner, Dinis Pedro Calado

**Affiliations:** 1 Immunity and Cancer, Francis Crick Institute, London, UK; 2 Immunoregulation, Francis Crick Institute, UK; 3 Bioinformatics and Biostatistics, Francis Crick Institute, London, UK; 4 Immune Receptor Activation Laboratory, Francis Crick Institute, London, UK; 5 Institute of Immunology and Immunotherapy, University of Birmingham, Birmingham, UK; 6 Theodor Boveri Institute and Comprehensive Cancer Center Mainfranken, Biocenter, University of Würzburg, Würzburg, Germany; 7 Division of Infection and Immunity, Institute of Immunity and Transplantation, University College London, London, UK

**Keywords:** IgG1, germinal center, B cell, positive selection, apoptosis, calcium, IP3 receptor, CRISPR-Cas9, Miz1 (Zbtb17), Tmbim4

## Abstract

The heavy chain isotype class confers specific properties to the antibody with critical consequences in infection and vaccination. For a robust class-switched antibody response, IgG1^+^ B cells outcompete IgM^+^ ones in germinal centers (GCs). Despite this knowledge the molecular mechanisms permitting IgG1^+^ GC B cell accruement remain unknown. Here we identified IgG1^+^ B cell transcription factor (TF) dependency using a CRISPR-Cas9 screen *in vitro* and mouse genetics *in vivo*. We found that the TF Miz1 (Zbtb17) was critically and specifically required for IgG1^+^ B cell survival during positive selection in GCs. Miz1 exerted this function through its target gene *Tmbim4*, that was required to prevent exacerbated IP3 receptor Ca^2+^ release during positive selection and protect IgG1^+^ GC B cells from death. These studies uncovered a protective mechanism permitting IgG^+^ GC B cell positive selection and provide an avenue to tailor isotype class specific GC responses in infection and vaccination.

## Graphical abstract

**Graphic abstract:**
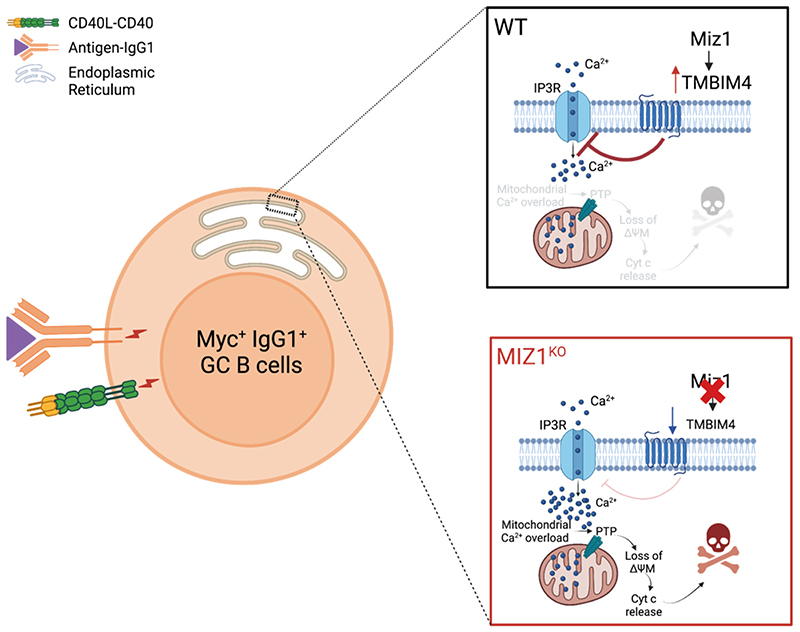

## Introduction

Durable humoral immune responses underly vaccination success [1]. The key process for an effective humoral immune response is the production of plasma cells (PCs) secreting diversified affinity matured antibodies towards invading pathogens. The heavy chain isotype class confers specific properties to the antibody. For example, μ heavy chains of IgM permit the formation of pentameric structures and are therefore strong complement activators [2, 3]. On the other hand the γ heavy chains of IgG, in addition to complement activation, are also effective in promoting immune cell-based cytotoxicity, phagocytosis and through the binding of Fcγ receptors primarily in innate cells, induce the release of inflammatory molecules [4].

PCs expressing affinity matured antibodies emerge from microstructures called germinal centers (GCs). GCs are normally formed in secondary lymphoid organs during an antigen and T cell dependent immune response [5]. GCs are composed by functionally and morphologically distinct regions, so-called light zone (LZ) and dark zone (DZ). In GCs, B cells undergo somatic hypermutation (SHM) in the DZ and those with higher affinity for the antigen that is presented as immune complexes by follicular dendritic cells (FDCs) in the LZ, are preferentially positively selected [6–10]. In addition to re-circulation in the GC, these positively selected GC B cells can differentiate into PCs and memory B cells (MBCs) [11–13]. These cellular GC outputs are critical for an effective immune response towards invading pathogens.

Current knowledge supports that fate determination of GC B cells to re-circulate in the reaction, or output as PCs or MBCs is not a random process [11, 12, 14]. In the LZ, GC B cells retrieve antigens from FDCs [15]. These antigens are processed and presented as pMHCII complexes to follicular helper T cells (TFHs) eliciting co-stimulation signals [16, 17]. GC B cells that acquired primary activation characterized by B cell receptor (BCR) engagement with antigen and secondary activation following TFH derived signals are called positively selected [5, 10, 18]. Myc expression is synergistically induced following primary and secondary activation and we and others have defined it as a marker of positive selection [18–20]. It is considered that GC B cell fate determination during positive selection occurs in an affinity-dependent manner [21–23]. GC B cells with relatively higher affinity can capture more antigen and present peptide-MHCII at higher density to TFHs, eliciting stronger co-stimulation signals. This results in higher Myc expression and mTORC1 signaling increasing cell cycle tempo and the number of proliferative rounds initially in the LZ and after that in the DZ [22, 24–26]. On other hand, GC B cells with the highest affinity commit preferentially to the PC fate [27], whereas those with relatively lower affinity are less effective in retrieving antigens, having weaker primary and secondary activation, becoming preferentially MBCs [26, 28–30].

More recently, we and others have proposed positive selection as a dynamic multi-layer biological process that allows for the retention of GC B cells with a range of affinities and thus being more complex than affinity maturity *per se* [26, 31–33]. Other work also supports that positive selection preferentially favors IgG1^+^ over IgM^+^ cells [34–36]. In this context, the knowledge that the heavy chain isotype class itself represents a positive selection layer was propelled by the finding that class switch recombination (CSR) primarily occurs before GC entry and is infrequent during GC reactions [34, 37]. Bias in positive selection favoring IgG1^+^ GC B cells is biologically consequential given that these cells preferentially commit to PC differentiation and are generally disfavored for the MBC fate [36]. Also, the production of affinity matured IgG1 is particularly vital for an effective defense against viral pathogens and for vaccination success [38–40]. However, we currently do not understand the molecular mechanisms permitting IgG1^+^ B cell accruement in GCs.

Here we used a CRISPR-Cas9 *in vitro* screen to identify IgG1^+^ B cell transcription factor (TF) dependency for survival and/or proliferation and found *Miz1* (*Zbtb17*) to be critically required. Notably, we previously showed that Miz1 is specifically expressed at high levels in GC B cells during positive selection [14]. Conditional *in vivo* inactivation of Miz1’s transcriptional activity reproduced the *in vitro* screen results. Specifically, we uncovered a previously unknown function for Miz1 in preventing exacerbated Ca^2+^ flux and as consequence protect IgG1^+^ GC B cells from death during positive selection. Miz1 exerted this function through Tmbim4, a newly described target of the TF. Tmbim4 mediated protection of IgG1^+^ GC B cells from death occurred through inhibition of exacerbated Ca^2+^ release via IP3 receptor (IP3R) during positive selection. This knowledge opens avenues to tailor isotype specific GC responses in infection and vaccination.

## Results

### An *in vitro* IgG1^+^ B cell CRISPR-Cas9 screen identifies *Miz1*

**Fig. 1:**
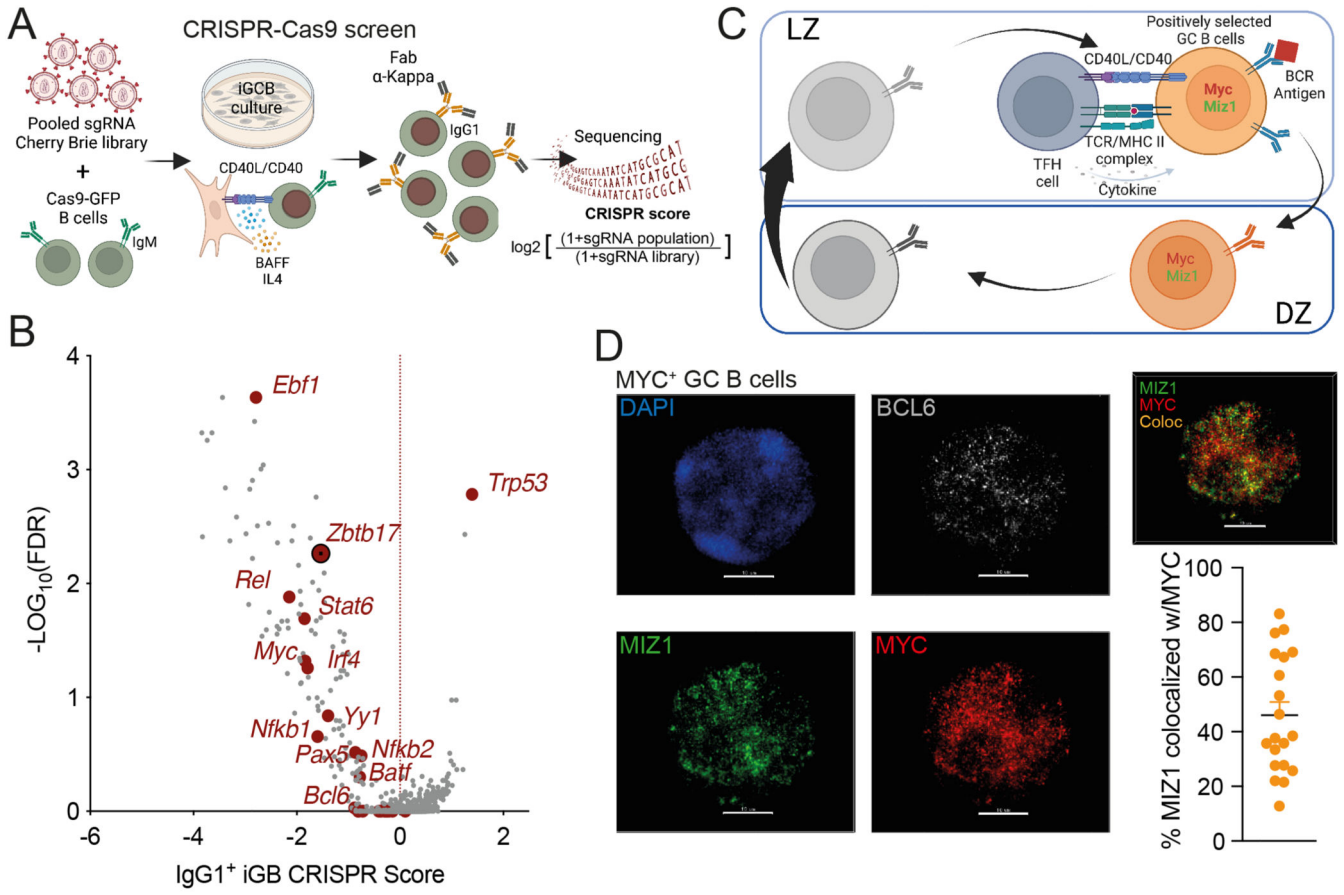
CRISPR screens identify *Miz1* as an essential TF in IgG1^+^ B cells. **(A)** Schematic of CRISPR screen workflow and IgG1^+^ analysis strategy. **(B)** Volcano plot of TF gene CRISPR scores versus statistical significance corrected for false discovery rate (FDR) for all TFs (gray) and essential TFs (dark red). **(C)** Schematic of Myc^neg^ LZ, Myc^pos^ LZ, Myc^pos^ DZ, Myc^neg^ DZ four consecutive GC B cell populations, and processes involved during positive selection. **(D)** Immunofluorescence stain of freshly isolated GC B cells. Left, representative counterstain of DAPI (blue), anti-Bcl6 antibody (grey), anti-Miz1 antibody (green) and anti-Myc antibody (red) in one GC B cell, scale bars = 20 μM. Right, distribution of Miz1 (green) and Myc (red) in the nucleus, colocalization of Miz1 and Myc is highlighted as orange = Colocalization. Graph represents the percentage of Miz1 co-localized with Myc in a single Myc^+^Miz1^+^ GC B cell. Each dot represents one cell (n=20), small horizontal line represents mean and SEM.

To identify candidate TFs required for IgG1^+^ GC B cell survival and/or proliferation we performed a CRISPR screen using *in vitro* derived GC B cells (iGB) [41, 42]. Genome-wide CRISPR screening was achieved by constructing the sgRNA Cherry-Brie library followed by infection of Cas9-expressing naïve mouse B cells [41, 43]. Transduced mouse B cells were then plated on the 40LB feeder cells with IL-4 to efficiently induce the generation of IgG1^+^ iGB cells. After 6 days of culture, we added anti-Igκ F(ab’)2 as antigen stimulation surrogate that together with CD40L, expressed by the 40LB feeder cells, mimicked the signals required for positive selection [18]. After 2 days, we FACS purified IgG1^+^ iGB cells and performed deep sequencing of sgRNAs to identify those that were depleted and to assign CRISPR scores to genes for which the loss impacted IgG1^+^ iGB cell survival and/or proliferation (Figure 1A). Among all essential TFs identified, Ebf1 ranked the highest (Figure 1B). This is consistent with knowledge that Ebf1 is required for GC B cell maintenance, and thus unlikely an isotype class specific effect [44]. The same applies to multiple other identified transcription TFs such as Myc, Bcl6, Pax5, Rel, Batf, Yy1 and Stat6 [19, 20, 45–55]. Nfkb1, Nfkb2, Irf4 are not essential for GC B cell maintenance; however, evidence supports that these TFs are required for CSR [56–61]. Because the original objective was to investigate mechanisms underlying IgG1^+^ B cell accruement in GCs, we focused on Miz1 (Zbtb17) that we have shown to be specifically expressed at high levels in positively selected i.e., Myc^+^ GC B cells *in vivo* [14] (Figure 1C). Myc and Miz1 are transcriptional activators; however, they can form transcriptional complexes that repress the expression of Miz1 target genes. However, our previous analysis of mice with impaired Myc-Miz1 complexes formation did not reveal altered IgG1^+^ B cell accruement in GCs [14]. This knowledge supports the hypothesis that Miz1 has functions in positively selected GC B cells beyond its interaction with Myc. Indeed, we found that on average 50% of Miz1 molecules did not colocalized with Myc in freshly isolated positively selected GC B cells (Figure 1D, S1, Supplemental Video 1).

### Loss of IgG1^+^ GC B cells upon Miz1 deficiency *in vivo*

**Fig. 2:**
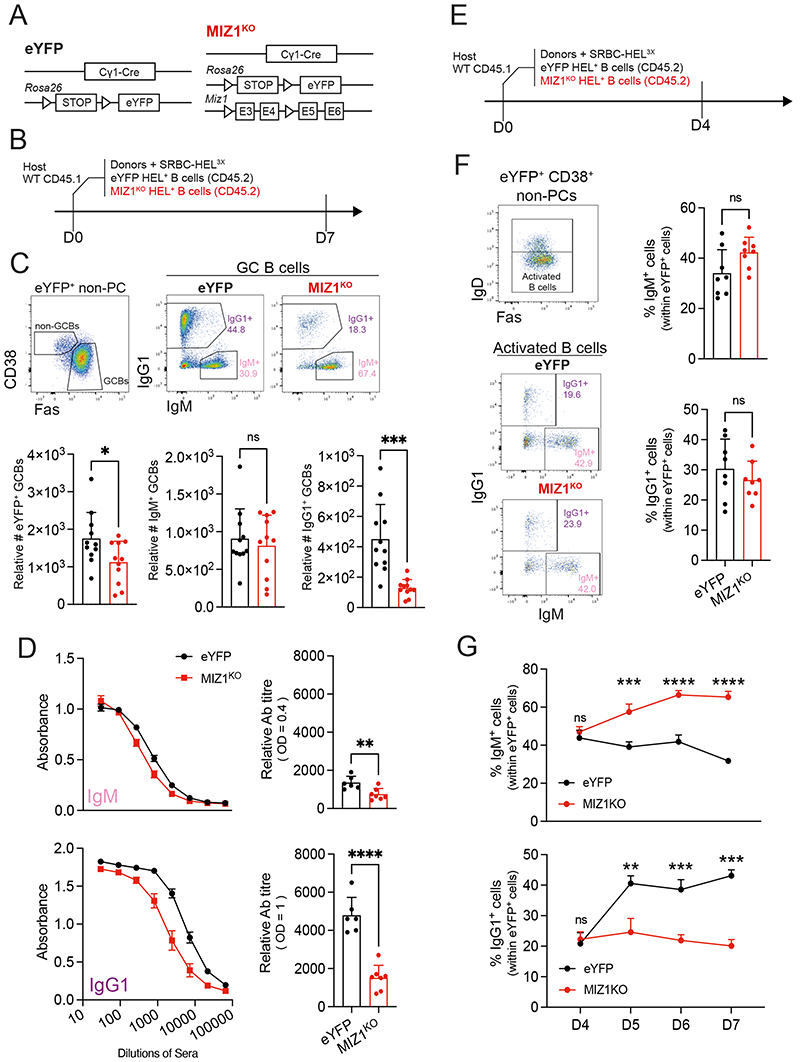
Miz1 loss impairs IgG1^+^ GC B cell accumulation *in vivo*. **(A)** Schematics of allele combination in control (eYFP) and experimental mice (MIZ1^KO^). An allele containing *loxP-stop-loxP-eYFP* (*loxP=* triangle) in the *Rosa26* locus is present in both eYFP and MIZ1^KO^ mice. MIZ1^KO^ contains an *Miz1* allele with two *loxP* (triangle) flanking exon3 and exon4 that encode the BTB/POZ DNA binding domain. **(B)** SWHEL experiment schematics. **(C)** Top, gating strategy for IgG1^+^ and IgM^+^ GC B cell subsets generated from donor-derived reporter positive B cells. Bottom, graphs displaying cumulative data of relative cell numbers (numbers per 10^6^ splenocytes) for total GC B cells, and GC B cell IgG1^+^ and IgM^+^ subsets in, eYFP (control, black); and MIZ1^KO^ (red). **(D)** Left, ELISA for HEL-binding IgG1 and IgM antibodies in recipient mice. Right, relative antibody titer of anti-HEL IgG1 (OD = 1), and anti-HEL IgM (OD = 0.4). **(E)** SWHEL experiment schematics as in (B). Recipient mice spleen and sera were analyzed at day 4 (F) after immunization. **(F)** Left, gating strategy; right, percentages of IgM^+^ and IgG1^+^ subsets within early activated B cells. **(G)** Dynamic changes of the percentages of IgG1^+^ and IgM^+^ B cell subsets in activated B cells (D4), and GC B cells (D5-7). Each symbol (C: eYFP n = 12, MIZ1^KO^ n = 12; D : eYFP n = 6, MIZ1^KO^ n = 7; F: day 4 eYFP n = 8, MIZ1^KO^ n = 8; H: day 4 eYFP n = 3, MIZ1^KO^ n = 3; day 5, 6 eYFP n = 6, MIZ1^KO^ n = 6; day 7 eYFP n = 3, MIZ1^KO^ n = 3) represents an individual mouse; small horizontal lines show mean and SEM. Data in (C-D) is representative of three to five experiments. Data in (F) is from two independent experiments. Data in (G), D4 and D7 is data from one experiment, D5-6 is data from two independent experiments. *, P ≤ 0.05; **, P ≤ 0.01; ***, P ≤ 0.001; ****, P ≤ 0.0001 (unpaired two-tailed Student’s t test in (C-D, F); multiple T tests in (G)). ns, not significant.

To investigate the function of Miz1 in positively selected GC B cells, we used *Cγ1-cre* [62] and a conditional allele composed by loxP flanking of the DNA region encoding the Miz1 POZ domain, critically required for transcriptional activity [63]. We further complemented the genetic system, hereafter referred to as MIZ1^KO^, with a R26 cre-loxP fluorescent reporter expressing eYFP (*R26eYFP^stopFL^*) [64], allowing to identify eYFP^+^ cells as surrogate of successful cre-mediated recombination. Mice carrying the C*γ1-cre* and *R26eYFP^stopFL^* alleles served as control and are hereafter referred as eYFP (Figure 2A). We also generated compound mutant mice that further carried the SWHEL system, in which B cells express a transgenic BCR recognizing hen egg lysozyme (HEL) [65]. According to established protocols [66] we adoptively transferred CD45.2^+^ HEL-specific B cells from donor mice into congenic CD45.1^+^ recipients, after which recipient mice were immunized with HEL^3X^ (Figure 2B). At day 7 following immunization we gated for GC B cells within eYFP^+^ cells and determined the numbers of IgM^+^ and IgG1^+^ B cells (Figure 2C). Ablation of Miz1’s transcriptional activity reduced the total number of GC B cells in accord with others [67], but had no impact on the number of IgM^+^ GC B cells. In contrast, MIZ1^KO^ displayed a remarkable reduction in the number of IgG1^+^ GC B cells, with less than 25% of cells remaining compared to control (Figure 2C). These data suggested that the transcriptional activity of Miz1 was critically required for normal numbers of IgG1^+^ B cells in GC reactions *in vivo*.

### Miz1 is required of IgG1^+^ B cell accruement in GCs

The observed reduction in the number of IgG1^+^ B cells in established GCs upon the loss of Miz1’s transcriptional activity could be the consequence of i) an increased output of cells as PCs or MBCs; ii) impaired class switching recombination (CSR) to IgG1 early on after immunization following IgM^+^ B cell activation; iii) a defect in positive selection of IgG1^+^ GC B cells.

To address the first possibility, we determined anti-HEL titers as a measure of antigen-specific PC abundance [68]. We found a remarkable reduction of anti-HEL IgG1 antibody titers in MIZ1^KO^ compared to eYFP control. In contrast, anti-HEL IgM antibody titers were only slightly reduced (Figure 2D). These data indicated that the output of IgG1^+^ PCs is severely decreased following the loss of Miz1’s POZ domain. Similarly, the numbers of IgG1^+^ MBCs in MIZ1^KO^ were significantly impaired compared to control (Figure S2A, B). We concluded that the observed reduction of IgG1^+^ GC B cell numbers in MIZ1^KO^ is not due to an increased GC output.

We next tested whether MIZ1^KO^ had CSR from IgM to IgG1 impacted. Given that CSR peaks before GC formation [37] we investigated the number of IgG1^+^ activated B cells early in the immune response i.e., before GC formation (Figure 2E). We gated on IgD^neg^ eYFP^+^ non-GC B cells to identify activated B cells and within these cells we defined the IgG1^+^ and IgM^+^ B cell subsets (Figure 2F). We found that the fraction of IgG1^+^ and IgM^+^ activated B cells was identical between MIZ1^KO^ and eYFP (Figure 2F). These data indicated that the reduced number of IgG1^+^ B cells in established GCs of MIZ1^KO^ was not due to impaired IgM to IgG1 CSR. To confirm this possibility, we amplified IgHγ1-germ line transcripts (GLTs) as an indicator of CSR from FACS purified naïve Follicular B cells, activated B cells and GC B cells (Figure S2C) [69]. Consistent with previous work [37], the amount of IgHγ-GLTs peaked in activated B cells, indicative of active CSR and these were not reduced in MIZ1^KO^ compared to control (Figure S2 D). Together these data support that CSR is not impaired in MIZ1^KO^.

As expected, the fraction of IgG1^+^ GC B cells greatly increased over time in GC reactions of the eYFP control (Figure 2G) [34]. However, this phenomenon was not observed in MIZ1^KO^, instead, the IgG1^+^ GC B cell fraction remained low with a trend for decrease over time (Figure 2G). A similar result was observed for IgM^neg^IgG1^neg^ GC B cells class-switched to other isotypes (Figure S2E). In contrast to IgG1^+^ GC B cells, the percentages of non-switched IgM^+^ GC B cells remained constantly higher in MIZ1^KO^ compared to eYFP control (Figure 2G). Thus, in the absence of an increased GC output or impaired CSR we considered that Miz1’s transcription activity is required for the positive selection of IgG1^+^ B cells in GC reactions.

### MIZ1^KO^ IgG1^+^ GC B cells undergo apoptosis during positive selection

**Fig. 3:**
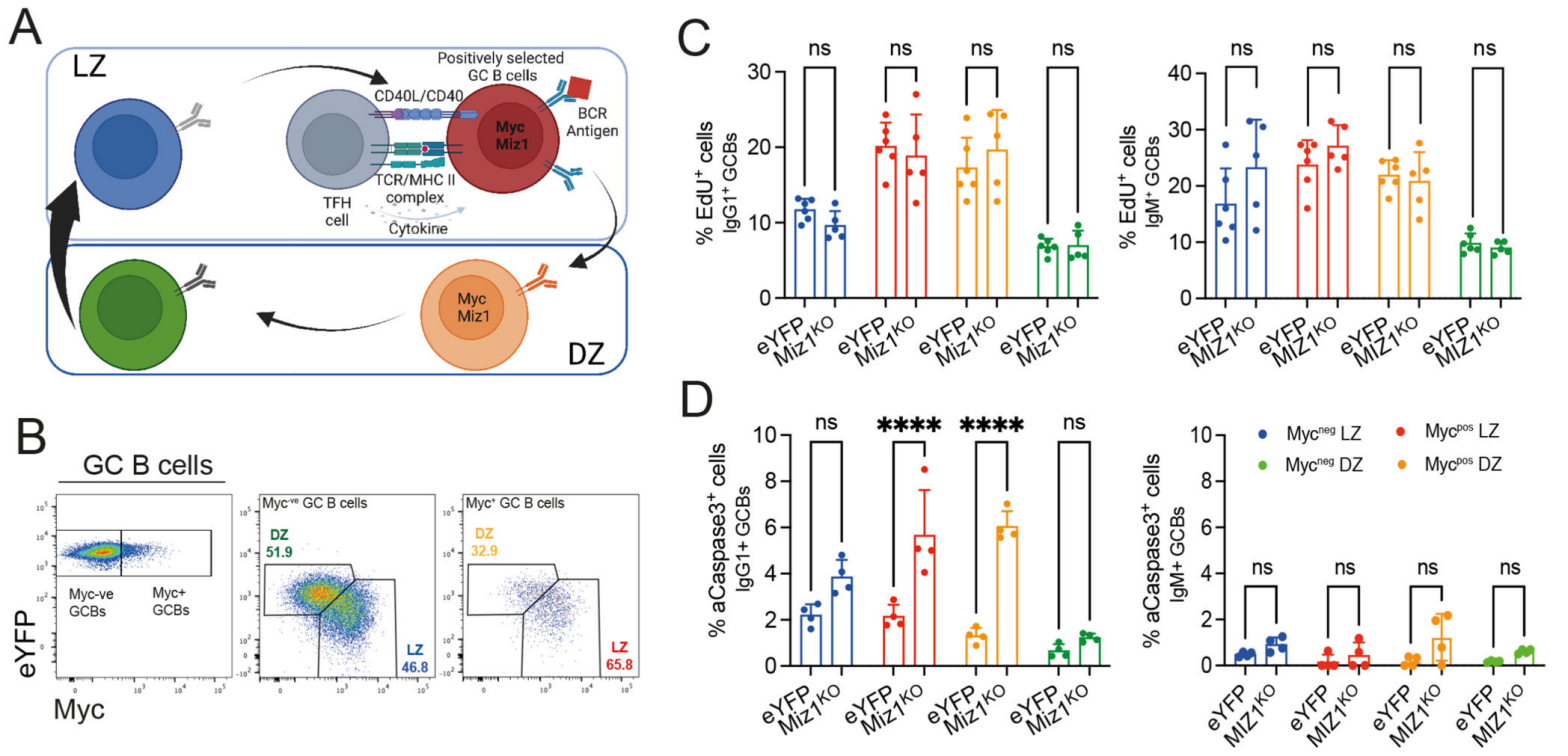
Miz1 is required for IgG1^+^ GC B cell survival during positive selection. **(A)** Schematic of Myc^neg^ LZ, Myc^pos^ LZ, Myc^pos^ DZ, Myc^neg^ DZ four consecutive GC B cell populations, and processes involved during positive selection. **(B)** Gating strategy for the four consecutive GC B cell populations as in (A). **(C)** Percentages of EdU^+^ cells within each of the four consecutive GC B cell populations in both eYFP and MIZ1^KO^ mice. **(D)** Percentages of active-Caspase3^+^ cells within each of the four consecutive GC B cell populations. Each symbol (C: eYFP n = 6, MIZ1^KO^ n = 6; D: eYFP n = 4, MIZ1^KO^ n = 4) represents an individual mouse; small horizontal lines show mean and SEM. Data in (C, D) is representative from two experiments. *, P ≤ 0.05; **, P ≤ 0.01; ***, P ≤ 0.001; ****, P ≤ 0.0001; Two-way ANOVA in (C, D)). ns, not significant.

To untangle the role of Miz1 for IgG1^+^ GC B cell positive selection, we investigated cell proliferation and survival. Our previous work and of others defined Myc as a marker of positively selected GC B cells [19, 20]. It is therefore possible to broadly resolve GC B cell dynamics by defining the reaction into four consecutive subpopulations i.e., Myc^neg^ LZ → Myc^pos^ LZ → Myc^pos^ DZ → Myc^neg^ DZ [26]. This increased resolution could also permit a better definition of Miz1’s function, because this TF is specifically highly expressed in positively selected cells i.e., Myc^+^ (Figure 3A, B) [14]. Next, we investigated cell proliferation using EdU incorporation. We found that the fraction of either EdU^+^ IgG1^+^ or EdU^+^ IgM^+^ GC B cells in each subpopulation was identical between MIZ1^KO^ and eYFP control (Figure 3C). We then tested for apoptosis using active-Caspase3 (aCaspase3^+^) intracellular stain and found a remarkably increased fraction of MIZ1^KO^ apoptotic IgG1^+^ GC B cells compared to eYFP (Figure 3D). Notably, this was only the case in the Myc^pos^ LZ and Myc^pos^ DZ subpopulations where there was more than a 3-fold increase in the fraction of apoptotic cells (Figure 3D). In contrast, we did not observe a difference in the apoptotic fraction across the four consecutive IgM^+^ GC B cell subpopulations in MIZ1^KO^ and eYFP mice (Figure 3D). Together, these data indicated that Miz1 is critically required to prevent apoptosis of IgG1^+^ GC B cells during positive selection.

### Miz1 regulates the expression of the anti-apoptotic factor Tmbim4

**Fig. 4:**
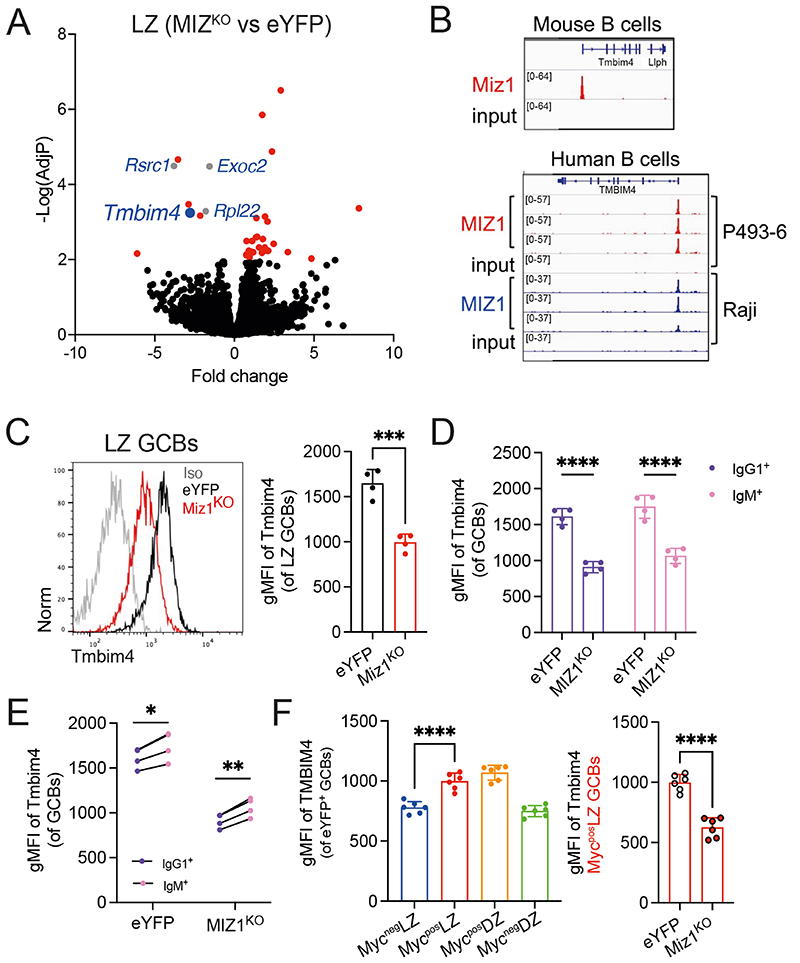
Miz1 regulates *Tmbim4* expression during positive selection. **(A)** Volcano plot for the fold change in gene expression and adjusted P value (-Log(AdjP)) between LZ GC B cells from MIZ1^KO^ mice versus LZ GC B cells from eYFP mice. Colored genes (Red, Blue, and Gray) have a -Log(AdjP) > 2. Genes in Gray are named and are reported Miz1’s target genes. Blue identifies *Timbim4*. **(B)** Top, Miz1 ChIP-seq data in mouse primary B cells for binding into the promoter region of the *Tmbim4* gene. Bottom, MIZ1 CHIP-seq data in human B cell lines (P493-6, Raji) for binding into the promoter region of the *TMBIM4* gene. **(C)** Left, representative plot for Tmbim4 expression levels in LZ GC B cells by intracellular stain. Right, cumulative data for Tmbim4 expression levels in LZ GC B cells, measured by gMFI. **(D)** Cumulative data for Tmbim4 expression levels in IgM^+^ and IgG1^+^ GC B cells, measured by gMFI. **(E)** Paired analysis for the expression of Tmbim4 in IgM^+^ and IgG1^+^ GC B cells derived from MIZ1^KO^ and eYFP control mice, measured by gMFI. **(F)** Left, cumulative data for Tmbim4 expression levels in Myc^neg^ LZ, Myc^pos^ LZ, Myc^pos^ DZ, Myc^neg^ DZ consecutive GC B cell populations from eYFP control mice. Right, comparison of Tmbim4 expression levels in Myc^pos^LZ GC B cells derived from MIZ1^KO^ and eYFP control mice. Each symbol (C-E: eYFP n = 4, MIZ1^KO^ n = 4; F: eYFP n = 6, MIZ1^KO^ n = 6) represents an individual mouse; small horizontal lines show mean and SEM. Data in (C-E) is representative of four experiments. **, P ≤ 0.01; ***, P ≤ 0.001; ****, P ≤ 0.0001 (unpaired two-tailed Student’s t test in (C, F); multiple paired t test in (E); Two-way ANOVA (D, F)).

To decipher mechanism/s underlying the role of Miz1 in IgG1^+^ GC B cells survival during positive selection we FACS purified LZ GC B cells from MIZ1^KO^ and eYFP control and investigated differentially expressed genes (DGEs) using RNA sequencing. We searched for pro-apoptotic genes fitting a criterion of <0.01 adjusted p-value and >2-fold change. The gene encoding the anti-apoptotic protein Tmbim4 [70] followed those criteria and was highly significantly downregulated in MIZ1^KO^ compared to eYFP; together with several previously reported Miz1 target genes, including Rpl22, Rscr1, Exoc2 [71–73] (Figure 4A). We investigated whether impaired expression of *Tmbim4* in Miz1^KO^ could be a direct consequence of the loss of Miz1 transcriptional activity. With this objective we generated and analyzed Miz1/MIZ1 chromatin immunoprecipitation sequencing (ChIP-seq) of mouse and human B cells, respectively. We found a specific and high enrichment of Miz1 at the promoter region of *Tmbim4* over input in mouse B cells, and this was confirmed for MIZ1 in two human B cell lines (Figure 4B). To test if impaired *Tmbim4* transcription impacted protein level, we stained GC B cells for Tmbim4 and found a clear reduction in LZ GC B cells from MIZ1^KO^ (Figure 4C). The loss of Miz1’s transcription ability resulted in reduced Tmbim4 expression in both IgG1^+^ and IgM^+^ GC B cells (Figure 4D). However, Tmbim4 expression level was still significantly higher in IgM^+^ GC B cells from MIZ1^KO^ compared to IgG1^+^ counterparts (Figure 4E). Reflecting the Miz1 expression pattern in GC B cell subpopulations we noticed that Tmbim4 was primarily induced from Myc^neg^ LZ → Myc^pos^ LZ in eYFP control. Indeed, the loss of Miz1’s transcription ability resulted in nearly 40% reduction of Tmbim4 expression in Myc^pos^ LZ GC B cells (Figure 4F). These data suggest that Miz1 controls the expression of the anti-apoptotic protein *Tmbim4*, a newly described target gene.

### Miz1 modulates Ca^2+^ release during positive selection

**Figure 5:**
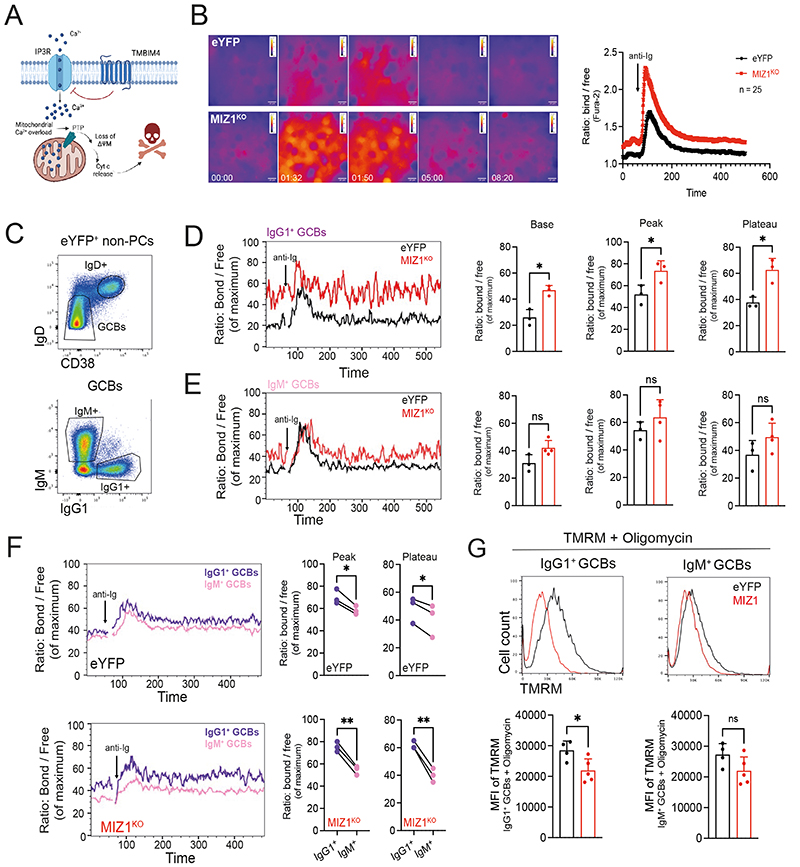
Miz1 limits Ca^2+^ release in IgG1^+^ GC B cells through Tmbim4. **(A)** Schematic diagram exemplifying the ER membrane, and TMBIM4 inhibition of Ca^2+^ release through modulation of IP3R. **(B)** Measurement of Ca^2+^ flux by live cell imaging. Fura-2 stained iGB cells were stimulated with anti-Ig antibody. Ratiometric images were generated by ImageJ. Left: time-point dynamics of Ca^2+^ flux before (00:00) and after stimulation (01:32, 01:50, 05:00 and 08:20). Right, analysis of ratio: bond / free Ca^2+^ reflecting cytoplasmic levels. Pooled data from 25 cells for each genotype. **(C)** Gating strategy of *ex-vivo* derived IgM^+^ and IgG1^+^ GC B cells subsets, for data presented in (D-E). **(D)** Left, representative Ca^2+^ flux dynamics calcium flux for Indo-1-stained IgG1^+^; right, graphs of cumulative data for “Base” = cytoplasmatic Ca^2+^ before stimulation, “Peak” and “Plateau” = cytoplasmatic levels of cytoplasmatic Ca^2+^ after stimulation. eYFP (control) black line, MIZ1^KO^ red line. **(E)** Left, representative Ca^2+^ flux dynamics calcium flux for Indo-1-stained IgM^+^; right, graphs of cumulative data as in (D). **(F)** Left, representative Ca^2+^ flux between IgG1^+^ (purple line) and IgM^+^ (pink line) GC B cells in each individual mouse of the same genotype. Right, “Peak” and “Plateau” = cytoplasmatic levels of cytoplasmatic Ca^2+^ after stimulation. Top, eYFP (control); bottom MIZ1^KO^. **(G)** Measurement of ΔΨ_M_ using TMRM dye. Top, representative TMRM levels from IgG1^+^ and IgM^+^ GC B cells derived *ex-vivo* from eYFP (control, black line) and MIZ1^KO^ (red line). Bottom, graphs displaying cumulative data analysis. MFI of TMRM is shown. Each symbol (D-F: eYFP n = 3, MIZ1^KO^ n = 3; G: eYFP n = 5, MIZ1^KO^ n = 5) represents an individual mouse; small horizontal lines show mean and SEM. Data in (B) is representative of four experiments. Data in (D-F) is representative of three experiments. Data in (G) is representative of two experiments. *, P ≤ 0.05; **, P ≤ 0.01 (unpaired two-tailed Student’s t test in (D, E, G); paired t test in (F)). ns, not significant.

Tmbim4 belongs to a newly identified anti-apoptotic TMBIM protein family [74]. TMBIM proteins are conserved across taxonomic kingdoms and are membrane-bond proteins expressed in most tissues and primarily localized on the surface of the Golgi apparatus and endoplasmic reticulum (ER) [70, 75, 76]. Previous work has shown that TMBIM4 modulates Ca^2+^ release from IP3 receptor (IP3R) at the ER [77] (Figure 5A). We therefore investigated if Ca^2+^ release is regulated downstream Miz1. In a first approximation we generated iGB cells using eYFP and MIZ1^KO^ naïve B cells and performed analysis at a stage of the culture in which more than 90% of cells had undergone CSR [42]. Stimulation of iGB cells with anti-Ig F(ab’)2 revealed an exacerbated Ca^2+^ flux in MIZ1^KO^ compared to eYFP, and the Ca^2+^ levels remained high in the former during the observation window (Figure 5B and Supplemental Video 2). We next performed *ex vivo* stimulation of freshly isolated GC B cells (Figure 5C). We found remarkably higher cytosolic Ca^2+^ levels in Miz1^KO^ IgG1^+^ GC B cells before and after stimulation of the BCR with anti-Ig F(ab’)2 compared to eYFP (Figure 5D). The basal, peak and plateau Ca^2+^ levels trended to be higher also in Miz1^KO^ IgM^+^ GC B cells compared to eYFP control, but this did not reach statistical significancy (Figure 5E). To better understand the impact of MIZ1^KO^ on Ca^2+^ release in an isotype specific manner, we compared IgM^+^ and IgG1^+^ GC B cells from each individual mouse. We found that IgG1^+^ GC B cells derived from eYFP displayed significantly higher levels of cytosolic Ca^2+^ compared to IgM^+^ GC B cells (Figure 5F). This finding was consistent with gene expression data [36] in which, compared to IgM^+^, IgG1^+^ GC B cells display enrichment for gene signatures associated with store operated Ca^2+^ entry (SOCE), Ca^2+^ release and import into cytosol, and mitochondrion (Figure S3).The loss of Miz1’s transcriptional activity led, however, to a striking increase in the release of Ca^2+^ in IgG1^+^ GC B cells compared IgM^+^ GC B cells in the same mouse (Figure 5F). In summary, these data support that IgG1^+^ GC B cells have increased Ca^2+^ flux during positive selection compared to IgM^+^, and that Miz1 functions to prevent exacerbation of this process.

An unchecked increase in cytosolic Ca^2+^ can trigger cell death [78]. During Ca^2+^ induced apoptosis, an inappropriately high cytosolic Ca^2+^ concentration triggers mitochondrial outer membrane permeabilization (MOMP) followed by the release of apoptogenic factors [79]. MOMP is directly associated with the loss of mitochondrial membrane potential (ΔΨ_M_) [80] (Figure 5A). Based on this knowledge, we tested the ΔΨ_M_ of IgG1^+^ GC B cells in both eYFP and MIZ1^KO^. We measured the *ex-vivo* ΔΨ_M_ of freshly isolated GC B cells and found that it was significantly compromised in MIZ1^KO^ IgG1^+^ GC B cells compared to eYFP control (Figure 5G). In contrast, the impact of the loss of Miz1’s transcriptional activity in the ΔΨ_M_ of IgM^+^ GC B cells was much less profound, not reaching statistical significance (Figure 5G). We conclude that Miz1 transcriptional activity modulates cytosolic Ca^2+^ levels and this is critical for the maintenance of ΔΨ_M_ in IgG1^+^ GC B cells.

### IP3R inhibition rescues ΔΨ_M_ and protects Miz1^KO^ GC B cells from apoptosis

**Fig. 6:**
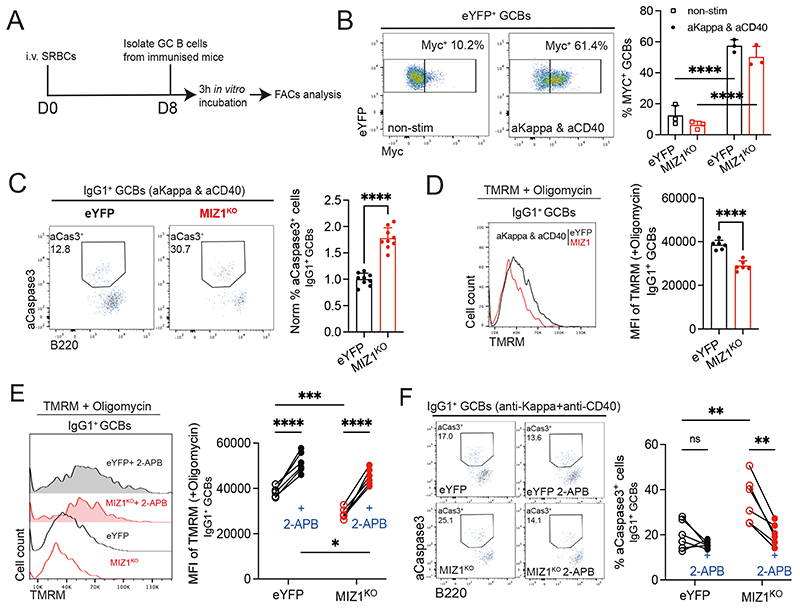
IP3R inhibition protects Miz1^KO^ IgG1^+^ GC B cells from apoptosis. **(A)** Schematics of the experimental workflow. **(B)** Left, Representative plots for the percentage of Myc^+^ GC B cells after 3 hours in culture under non-stimulation condition (non-stim) and upon F(ab’)2 anti-Kappa and anti-CD40 antibody stimulation (aKappa & aCD40). Right, graph displaying representative data for eYFP (control, black line) and MIZ1^KO^ (red line). **(C)** Measurement of apoptosis by active-Casepase3^+^ stain. Left, representative gating strategy for active-Casepase3^+^ (aCaspase3) cells by intracellular stain of IgG1^+^ GC B cells upon 3-hour stimulation with F(ab’)2 anti-Kappa and anti-CD40 antibody (aKappa & aCD40). Right, graph displaying cumulative data of normalized percentages of active-Casepase3^+^ IgG1^+^ GC B cells. eYFP (control, black line) and MIZ1^KO^ (red line). **(D)** Measurement of ΔΨ_M_ using TMRM dye. Left, representative TMRM levels from IgG1^+^ GC B cells derived from *in vitro* cultures as in (C). Right, graph displaying cumulative data analysis. MFI of TMRM is shown. **(E)** Impact of IP3R inhibitor 2-APB on ΔΨ_M_. Left, representative TMRM levels from IgG1^+^ GC B cells derived from *in vitro* cultures as in (C) treated (filled plots) or not (single line) with 2-APB. Right, graph displaying cumulative data paired analysis. MFI of TMRM is shown. **(F)** Impact of IP3R inhibitor 2-APB on apoptosis. Left, representative gating strategy for active-Casepase3^+^ (aCaspase3) in IgG1^+^ GC B cells from *in vitro* cultures as in (C) treated (filled plots) or not (single line) with 2-APB. Right, graph displaying cumulative data of percentages of active-Casepase3^+^ IgG1^+^ GC B cells. Each symbol (B: eYFP n = 3, MIZ1^KO^ n = 3; C: eYFP n = 9, MIZ1^KO^ n = 9; D-E: eYFP n = 6, MIZ1^KO^ n = 6; F: eYFP n = 6, MIZ1^KO^ n = 6) represents an individual mouse; small horizontal lines show mean and SEM in (C, E). Data in (B) is representative of three experiments. Data in (C) is from three independent experiments. Data in (D-E) is representative of two experiments. Data in (F) is from two independent experiments. *, P ≤ 0.05; **, P ≤ 0.01; ***, P ≤ 0.001; ****, P ≤ 0.0001 (unpaired two-tailed Student’s t test (C, D), Two-way ANOVA (B, E, F)).

We mimicked positive selection in *ex vivo* freshly isolated GC B cells using anti-Igκ plus anti-CD40 stimulation (Figure 6A) [18]. In agreement, this condition led to the highest Myc induction, compared to the simulation with either anti-Igκ or anti-CD40 stimulation alone (Figure 6B and Figure S4A). We next determined the fraction of aCaspase3^+^ cells and found that anti-Igκ plus anti-CD40 stimulation greatly reduced cell death of IgG1^+^ GC B cells derived from eYFP control (Figure S4B). However, and in contrast, this stimulus failed to rescue MIZ1^KO^ IgG1^+^ GC B cells from apoptosis (Figure 6C and Figure S4B). To directly test if increased cell death of MIZ1^KO^ IgG1^+^ GC B cells was the result of uncontrolled IP3R Ca^2+^ release [81], we used the IP3R antagonist 2-APB [82]. *Ex vivo* stimulated MIZ1^KO^ IgG1^+^ GC B cells displayed a profound reduction of ΔΨ_M_ compared to eYFP, confirming impaired mitochondrial function (Figure 6D). However, inhibition of IP3R Ca^2+^ release by 2-APB effectively rescued the ΔΨ_M_ of MIZ1^KO^ IgG1^+^ GC B cells (Figure 6E) and prevented the increased cell death observed in these cells compared to eYFP (Figure 6F). In contrast to *in vivo*, under *ex-vivo* supraphysiological stimulation conditions the loss of Miz1’s transcriptional activity also impacted, albeit in a less pronounced manner, the survival of IgM^+^ GC B cells (Figure S4C), and such was rescued by 2-APB (Figure S4D-E), Together these data supported that exacerbated Ca^2+^ release from IP3R underlies increased cell death upon loss of Miz1’s transcription capacity.

### Tmbim4 restoration rescues *in vivo* positive selection of MIZ1^KO^ IgG1^+^ GC B cells

**Fig. 7:**
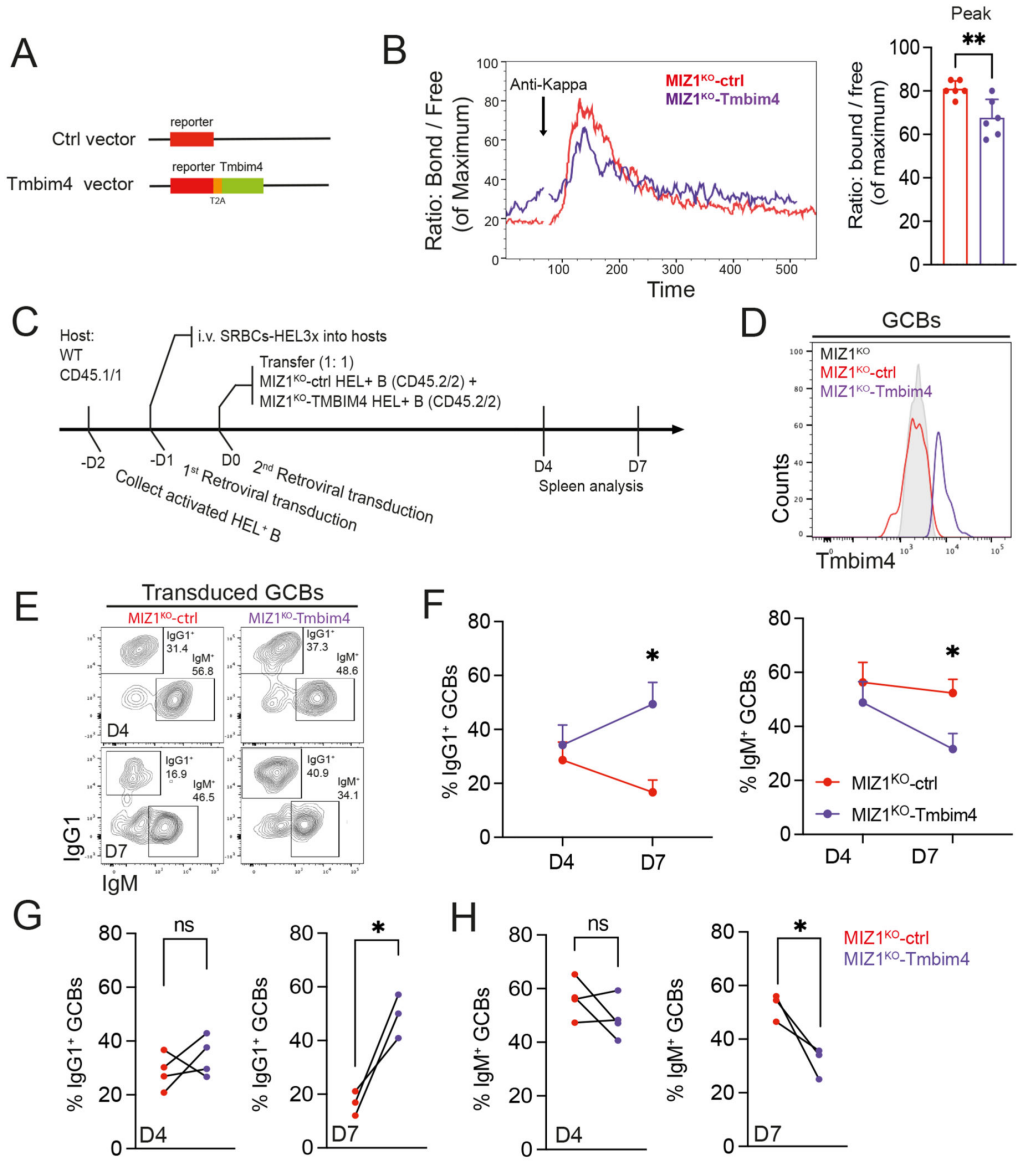
Tmbim4 restoration rescues IgG1^+^ GC B cell positive selection. **(A)** Schematic design of the retroviral vectors for Tmbim4 restoration and control. **(B)** Left, representative Ca^2+^ flux dynamics for Indo-1 stained iGB cells upon restoration (Miz1^KO^-Tmbim4) or not (Miz1^KO^-ctrl) of Tmbim4 in MIZ1^KO^ cells. Right, cumulative data for Ca^2+^ flux shown as “Peak” = highest cytoplasmatic levels after stimulation. **(C)** Schematics of the experimental workflow for the restoration of Tmbim4 levels *in vivo*. **(D)** Representative plots of *ex vivo* GC B cells displaying restoration (Miz1^KO^-Tmbim4) or not (Miz1^KO^-ctrl) of Tmbim4. **(E)** Gating strategy for IgG1^+^ and IgM^+^ GC B cell subsets generated from donor-derived reporter positive B cells. **(F)** Dynamic changes of the percentages of IgG1^+^ and IgM^+^ B cell subsets in retrovirally transduced GC B cell on day 4 and day 7. **(G)** Cumulative data for IgG1^+^ B cell percentages within GC B cells upon restoration (Miz1^KO^-Tmbim4) or not (Miz1^KO^-ctrl) of Tmbim4 in MIZ1^KO^ cells. Left, day 4 after cell transfer; right, day 7 after cell transfer. **(H)** Cumulative data for IgG1^+^ B cell percentages within GC B cells upon restoration (Miz1^KO^-Tmbim4) or not (Miz1^KO^-ctrl) of Tmbim4 in MIZ1^KO^ cells. Left, day 4 after cell transfer; right, day 7 after cell transfer. Each symbol (B: MIZ1^KO^-ctrl n = 6, MIZ1^KO^-Tmbim4 n = 6; E-H: day 4 n = 4; day 7 n = 3) represents an individual mouse. Data in (B) is from two independent experiments, data in (E - F) is one representative from two independent experiments. *, P ≤ 0.05; **, P ≤ 0.01 (unpaired two-tailed Student’s t test in (B); paired multiple t test (F); paired t test (G-H)).

To investigate if the increased Ca^2+^ release was a direct consequence of impaired Tmbim4 expression we restored Tmbim4 expression in MIZ1^KO^ iGC B cells and performed analysis of Ca^2+^ flux at a stage of the culture in which more than 80% of cells had undergone CSR (Figure 7A). By detecting RFP^+^ retrovirally transduced iGB cells, we found that Tmbim4 restoration effectively reduced MIZ1^KO^ induced Ca^2+^ release (Figure 7B). Together these data showed that restoration of Tmbim4 compensates the loss of Miz1’s transcriptional activity by moderating Ca^2+^ flux.

We next tested if the restoration of Tmbim4 expression in MIZ1^KO^ GC B cells rescued *in vivo* IgG1^+^ GC B cell positive selection. Using established protocols [83] we adoptively transferred equal numbers of control reporter vector and Tmbim4 vector (Figure 7A) transduced MIZ1^KO^ HEL^+^ B cells into the same recipient mouse followed by HEL^3X^ immunization (Figure 7C). MIZ1^KO^ GC B cells carrying the Tmbim4 vector displayed, as expected, elevated Tmbim4 expression compared to those carrying the control vector (Figure 7D). We then analyzed the transduced MIZ1^KO^ GC B cells at day 4 after immunization, representing early formed GCs in this system [68], and at 7 days after immunization when GCs had matured (Figure 7E). As expected, the fraction of control vector transduced MIZ1^KO^ IgG1^+^ GC B cells decreased overtime between day 4 and day 7 of the GC reaction (Figure 7F, G), confirming impaired ability of IgG1^+^ GC B cell positive selection. In contrast, Timbm4 restoration rescued positive selection of IgG1^+^ over IgM^+^ GC B cells in the absence of Miz1’s transcriptional activity, as reflected by a decreased fraction of IgM^+^ and increased fraction of IgG1^+^ GC B cells at day 7 (Figure 7F, H). These data indicates that positive selection of IgG1^+^ GC B cells requires a protective mechanism mediated by Miz1 → Tmbim4 to prevent exacerbated IP3R Ca^2+^ release and cell death.

## Discussion

The heavy chain isotype class confers unique properties to the antibody [2–4] that critically impact the quality and effectiveness of the humoral immune response to infection and in vaccination [38–40]. In GCs IgG1^+^ outcompete IgM^+^ B cells leading to the accruement of the former overtime. This bias is biologically consequential given that IgG1^+^ GC B cells preferentially commit to PC differentiation [36]. However, the molecular mechanisms permitting IgG1^+^ B cell accruement in GCs remained unknown.

Here, we showed that positive selection of IgG1^+^ GC B cells can only be achieved in the presence of intact Miz1 transcriptional activity. In this context, Miz1 directly upregulated the expression of the anti-apoptotic protein Tmbim4 at the transition between Myc^neg^ LZ to Myc^pos^ LZ stages i.e., during positive selection, to prevent exacerbated Ca^2+^ release by IP3R. This mechanism protected IgG1^+^ GC B cells from Ca^2+^ induced cell death. The occurrence of Ca^2+^ fluxes in GC B cells is supported by a recent study using intravital imaging and a novel reporter mouse analyzing Ca^2+^ release after GC B cell engagement with antigens [84]. The capability of exacerbated Ca^2+^ to induce GC B cell death agrees with the phenotypes observed upon excessive or hyperactive BCR signaling [85, 86] . Thus, and despite the Ca^2+^ flux of GC B cells being weaker than that of naïve B cells [78], the current study demonstrates the need for a tight regulation of Ca^2+^ release during positive selection of IgG1^+^ GC B cells. These findings provide new and unexpected insights into heavy chain isotype class dependent positive selection.

A previous study showed that IgG1^+^ GC B cells are favored during positive selection over IgM^+^, a phenomenon attributed to structural properties of γ heavy chains [34]. Despite the structure of IgG1 being intact in Miz1^KO^, IgG1^+^ GC B cells failed to accumulate overtime. These observations support a multi-layer scenario in which structural properties of γ heavy chains function together with an anti-apoptotic protective mechanism mediated by Miz1 → Tmbim4 for IgG1 positive selection to ensue. The structural features of γ heavy chains, such as hinge domains, were shown to impart more flexibility and stronger avidity for IgG1 to engage with the antigen displayed on FDCs [87–89]. These γ heavy chains properties likely induce stronger and longer-lasting Ca^2+^ release in IgG1^+^ GC B cells. This is supported by early experiments using naïve B cells and cell-lines in which compared to IgM, IgG1 initiates stronger and longer-lasting Ca^2+^ mobilization upon stimulation [90, 91]. In this study we showed that compared to IgM^+^ counterparts, IgG1^+^ GC B cells also mediated stronger Ca^2+^ release. In the GC context, however, Miz1 transcriptional deficiency remarkably exacerbates this difference (Figure 5F). Stronger Ca^2+^ signal following antigen response may underlie, at least in part, the advantage of IgG1^+^ over IgM^+^ GC B cells during positive selection [90–93]. However, the current work demonstrates that in GCs the enhanced ability of IgG1 to mobilize Ca^2+^ is a double-edged sword that must be finely tuned to prevent cell death.

The current study focused on IgG1^+^ class switched GC B cells. We noticed, however, that other class switched GC B cells (IgM^neg^IgG1^neg^) were similarly impacted in Miz1^KO^ (Figure S2E), with their cell numbers decreasing overtime during GC responses. Notably, loss of Miz1’s transcriptional activity had no negative impact on the quantity of IgM^+^ GC B cells (Figure 2). This is in agreement with a significantly higher Tmbim4 expression in IgM^+^ GC B cells compared their IgG1^+^ counterparts (Figure 4E). Miz1^KO^ resulted only in a small increase of cytosolic Ca^2+^ (Figure 5E) and no impact was observed on IgM^+^ GC B cells positive selection *in vivo.* Nevertheless, the protective mechanism formed by Miz1 → Tmbim4 is functional in IgM^+^ GC B cells because in contrast to *in vivo*, under *ex-vivo* supraphysiological stimulation conditions we observed a small but significant increase in cell death of MIZ1^KO^ IgM^+^ GC B cells.

Miz1 is specifically highly expressed in positively selected GC B cells i.e., Myc^+^ [14]. Both Myc and Miz1 are transcription activators [94, 95], but can form transcriptional complexes that repress the expression of Miz1 target genes [96–98]. Previously we found that the formation of these complexes is required to drive cell cycle engagement of positively selected GC B cells [14]. Here we showed that Miz1 by inducing *Tmbim4* moderates Ca^2+^ mobilization (Figure 4). This cumulative knowledge establishes Miz1 as crucial TF in the GC reaction with functions consistent with that of a negative regulator of positive selection signals.

## Limitations

During positive selection Miz1 → Tmbim4 prevents exacerbated Ca^2+^ mobilization and cell death of IgG1^+^ GC B cells. Although we used multiple immunization conditions, monoclonal and polyclonal mice, it will be relevant to study whether different infectious agents can modulate Miz1 levels and activity. We also have not explored in detail the role of Miz1 beyond IgG1 GC B cells. Thus, whether Miz1 → Tmbim4 is required to prevent cell death of GC B cells carrying other isotypes, namely IgA and IgE remains to be investigated.

## Supplementary Material

Data file S1

Supplemental Video 1

Supplemental Video 2

Supplemental Video 3

Supplementary Material

## Highlight

1. Identification of IgG1^+^ B cell transcription factor dependency using a CRISPR screen.
2. The transcription factor Miz1 (Zbtb17) is required for IgG1^+^ GC B cell survival.
3. The Miz1 target gene Tmbim4 moderates Ca^2+^ release to prevent IgG1^+^ GC B cell death.
4. Tmbim4 operates through IP3 receptors permitting IgG1^+^ GC B cell positive selection.
