# Supplementary material for: Regulation of BCR-mediated Ca2+ mobilization by MIZ1-TMBIM4 safeguards IgG1+ GC B cell-positive selection
Source: Sci Immunol. Author manuscript; Available in PMC 2024 Apr 29. (PMC7615907; doi:10.1126/sciimmunol.adk0092)
Supplement: Supplementary Material [file EMS195667-supplement-Supplementary_Material.pdf]

Supplemental Figure Legends

Video S1

Representative three-dimensional imaging of Miz1 and Myc colocalization in positively selected (Myc^{+}) GC B cells. Nucleus was identified with DAPI (blue) staining, and counterstained with Bcl-6 (grey), Miz1 (green) and Myc (red). Imaging was generated by Imaris software.

Fig. S1 (Related to Fig. 1)

3D image analysis of positively selected GC B cells for Miz1 and Myc colocalization.

GC B cells counterstained with DAPI (blue), anti-Bcl-6 antibody (grey), anti-Miz1 antibody (green) and anti-Myc antibody (red). Data analyzed using Imaris software.

Fig. S2 (Related to Fig. 2)

Normal CSR but impaired $IgG1^{neg}$ IgM^{neg} GC B cell accruement and $IgG1^{+}$ MBC formation *in vivo*

(A) Gating strategy of $eYFP^{+}$ MBCs, $IgG1^{+}$ and IgM^{+} MBCs subsets.

(B) Relative cell numbers (numbers per 10^6 splenocytes) of $eYFP^{+}$ MBCs, $IgG1^{+}$ and IgM^{+} MBCs subsets.

(C) Representative sorting strategy to isolate Follicular B cells (Fo), activated B cells (Activ-B), and GC B cells (GCB).

(D) RT-PCR analysis of sorted B cells as in (D) for γ1-GLT expression. eYFP (control, black); and $MIZ1^{KO}$ (red).

(E) Top, gating strategy for $IgG1^{neg}IgM^{neg}$ B GC B cells generated from donor-derived reporter positive B cells. Bottom, graphs displaying cumulative data of percentages for $IgG1^{neg}IgM^{neg}$ activated B cells (D4) and GC B cells (D5-7), eYFP (control, black); and $MIZ1^{KO}$ (red).

Each symbol (B: eYFP n = 9, $MIZ1^{KO}$ n = 9; C: Fo eYFP n = 2, $MIZ1^{KO}$ n = 2; Acti-B eYFP n = 6, $MIZ1^{KO}$ n = 3; GCB eYFP = 3, $MIZ1^{KO}$ n = 3; D: day 4 eYFP n = 3, $MIZ1^{KO}$ n = 3; day 5 eYFP n = 6, $MIZ1^{KO}$ n = 6; day 7 eYFP n = 3, $MIZ1^{KO}$ n = 3;) represents an individual mouse; small horizontal lines show mean and SEM. Data in (B) is from three independent experiments. Data in (C) D4 and D7 is data from one experiment, D5-6 is data from two independent experiments. Data in (E) is from two experiments. *, $P \leq 0.05$; **, $P \leq 0.01$; ***, $P \leq 0.001$; ****, $P \leq 0.0001$ (unpaired two-tailed Student's t test (B); multiple t test (C, E).

Video S2 (Related to Figure 5)

Dynamic measurement of Ca^{2+} flux by live cell imaging. Fura-2 stained iGB cells were stimulated with anti-Ig antibody. Ratiometric video images were generated by ImageJ.

Fig. S3 (Related to Figure 5)

IgG1^{+} GC B cells mediate increased Ca^{2+} flux compared to IgM^{+}.

Gene set enrichment analysis (GSEA) of differentially expressed genes (DEGs) in LZ IgG1^{+} versus LZ IgM^{+} GC B cells for signatures associated with store operated Ca^{2+} entry (SOCE), Ca^{2+} release, Ca^{2+} import into cytosol and mitochondrion. FDR, false discovery rate; NES, normalized enrichment score. The list of DEGs was generated from the analysis of RNAseq data in GSE76864.

Fig. S4 (Related to Figure 6)

IP3R inhibition protects MIZ1^{KO} GC B cells from apoptosis.

(A) Left, representative plots and percentages of Myc^{+} cells within GC B cells from different *in vitro* stimulation conditions for 3 hours. Right, cumulative data of the analysis performed as in (A) for eYFP (control) and MIZ^{KO} mice. Non-stimulation condition (non-stim, gray); F(ab')2 anti-Kappa (aKappa, green), anti-CD40 antibody (aCD40, orange), F(ab')2 anti-Kappa and anti-CD40 antibody (aKappa & aCD40, purple).

(B) Representative gating and percentages of active-Caspase3^{+} (aCasp3^{+}) cells within IgG1^{+} GC B cells from different *in vitro* stimulation conditions for 3 hours. Right, cumulative data of the analysis performed as in (A) for eYFP (control) and MIZ^{KO} mice. Non-stimulation condition (non-stim, gray); F(ab')2 anti-Kappa (aKappa, green), anti-CD40 antibody (aCD40, orange), F(ab')2 anti-Kappa and anti-CD40 antibody (aKappa & aCD40, purple).

(C) Normalized percentage of active-Casepase3^{+} (a-Casepase3^{+}) cells in *in vitro* F(ab')2 anti-Kappa and anti-CD40 antibody stimulated IgM^{+} GC B cells.

(**D**) Impact of IP3R inhibitor 2-APB on $\Delta\Psi_M$ of *in vitro* F(ab')2 anti-Kappa and anti-CD40 antibody stimulated IgM^{+} GC B cells.

(**E**) Impact of IP3R inhibitor 2-APB on apoptosis of *in vitro* F(ab')2 anti-Kappa and anti-CD40 antibody stimulated IgM^{+} GC B cells.

Each symbol (A-B: eYFP n = 3, MIZ1^{KO} n = 3; C: eYFP n = 9, MIZ1^{KO} n = 9; D-E: eYFP n = 6, MIZ1^{KO} n = 6) represents an individual mouse; small horizontal lines show mean and SEM

in (A, B, C). Data in (A-B) is representative data from three independent experiments. Data in (C) is from three independent experiments. Data in (D) is one representative from two independent experiments. Data in (E) is from two experiments. *, $P \leq 0.05$; **, $P \leq 0.01$; ***, $P \leq 0.001$; ****, $P \leq 0.0001$ (Two-way ANOVA (D, E)). ns, not significant.

Figure S1

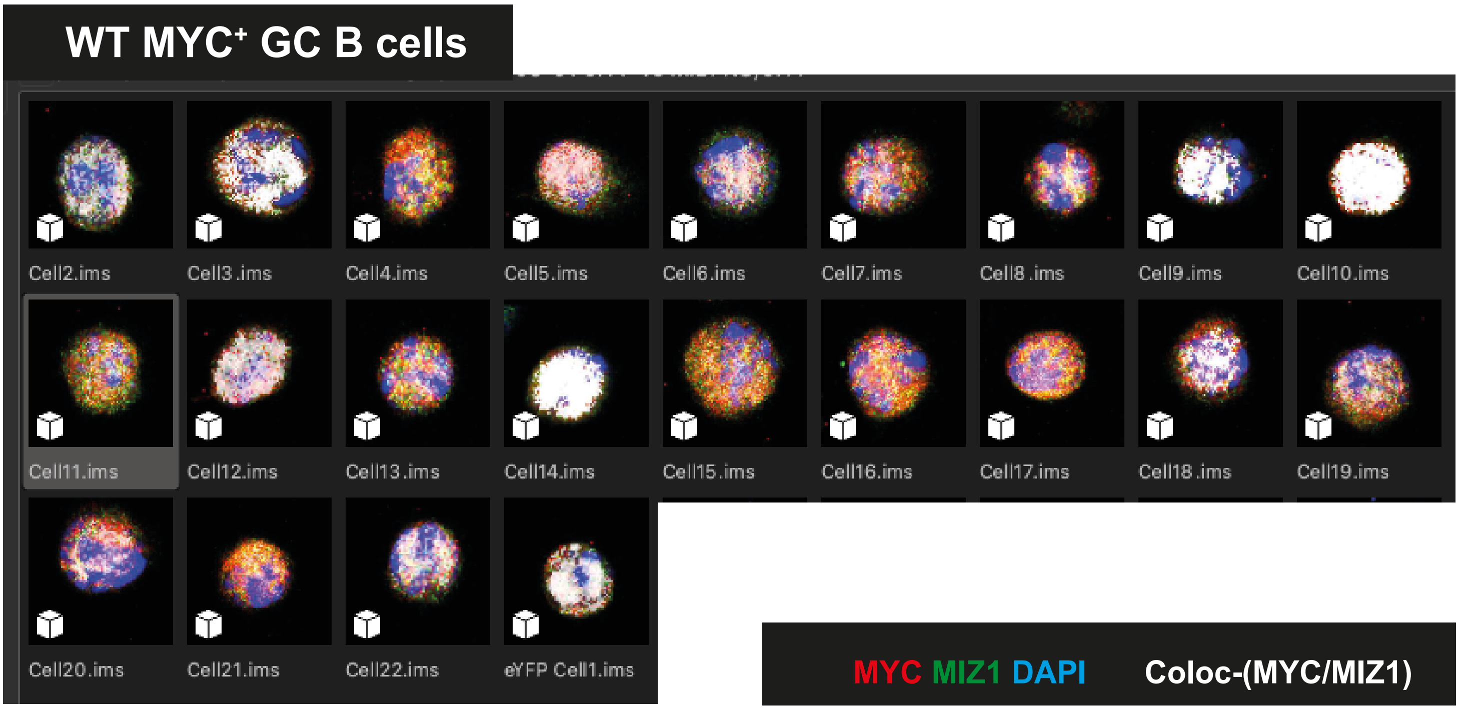
WT MYC+ GC B cells
Cell2.ims
Cell3.ims
Cell4.ims
Cell5.ims
Cell6.ims
Cell7.ims
Cell8.ims
Cell9.ims
Cell10.ims
Cell11.ims
Cell12.ims
Cell13.ims
Cell14.ims
Cell15.ims
Cell16.ims
Cell17.ims
Cell18.ims
Cell19.ims
Cell20.ims
Cell21.ims
Cell22.ims
eYFP Cell1.ims
MYC MIZ1 DAPI
Coloc-(MYC/MIZ1)

Figure S2

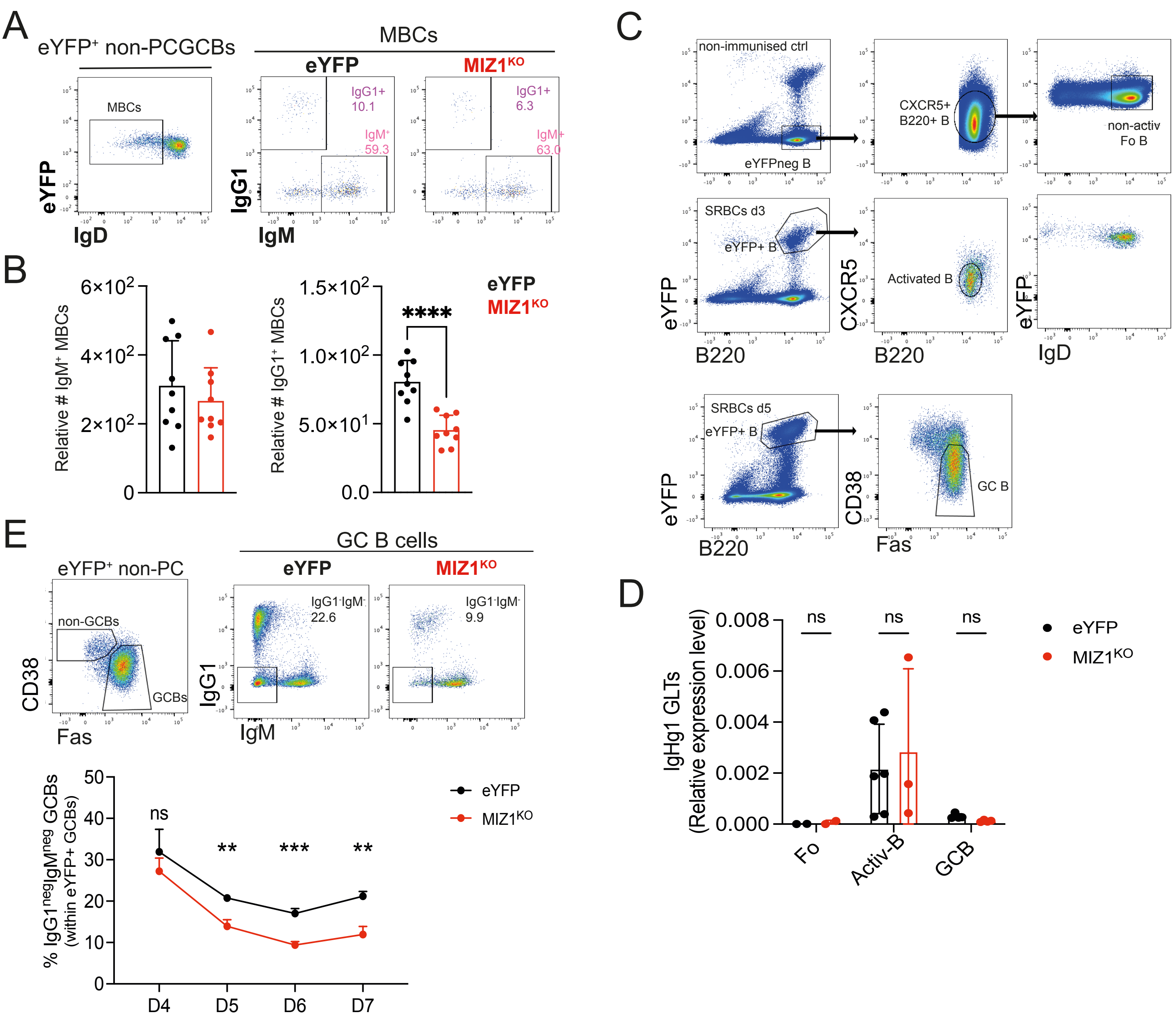

Zhang, et al.

Figure S3

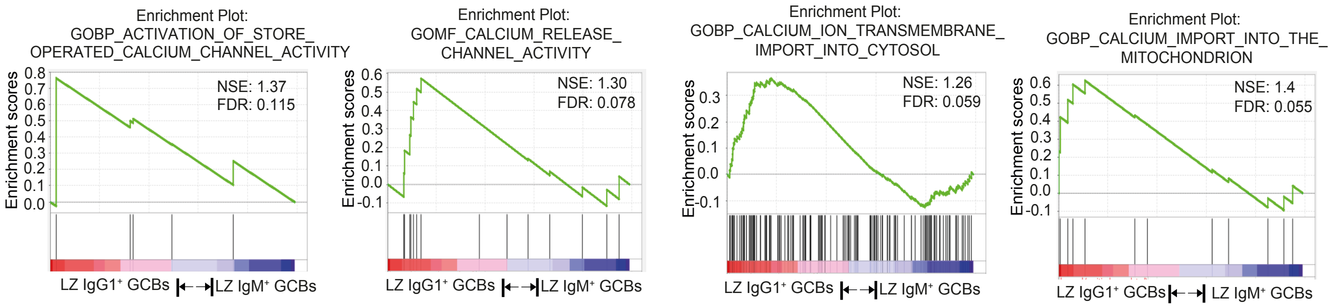
Enrichment Plot:
GOBP_ACTIVATION_OF_STORE_
OPERATED_CALCIUM_CHANNEL_ACTIVITY
NSE: 1.37
FDR: 0.115
Enrichment scores
LZ IgG1+ GCBs
LZ IgM+ GCBs
Enrichment Plot:
GOMF_CALCIUM_RELEASE_
CHANNEL_ACTIVITY
NSE: 1.30
FDR: 0.078
Enrichment scores
LZ IgG1+ GCBs
LZ IgM+ GCBs
Enrichment Plot:
GOBP_CALCIUM_ION_TRANSMEMBRANE_
IMPORT_INTO_CYTOSOL
NSE: 1.26
FDR: 0.059
Enrichment scores
LZ IgG1+ GCBs
LZ IgM+ GCBs
Enrichment Plot:
GOBP_CALCIUM_IMPORT_INTO_THE_
MITOCHONDRION
NSE: 1.4
FDR: 0.055
Enrichment scores
LZ IgG1+ GCBs
LZ IgM+ GCBs

Figure S4

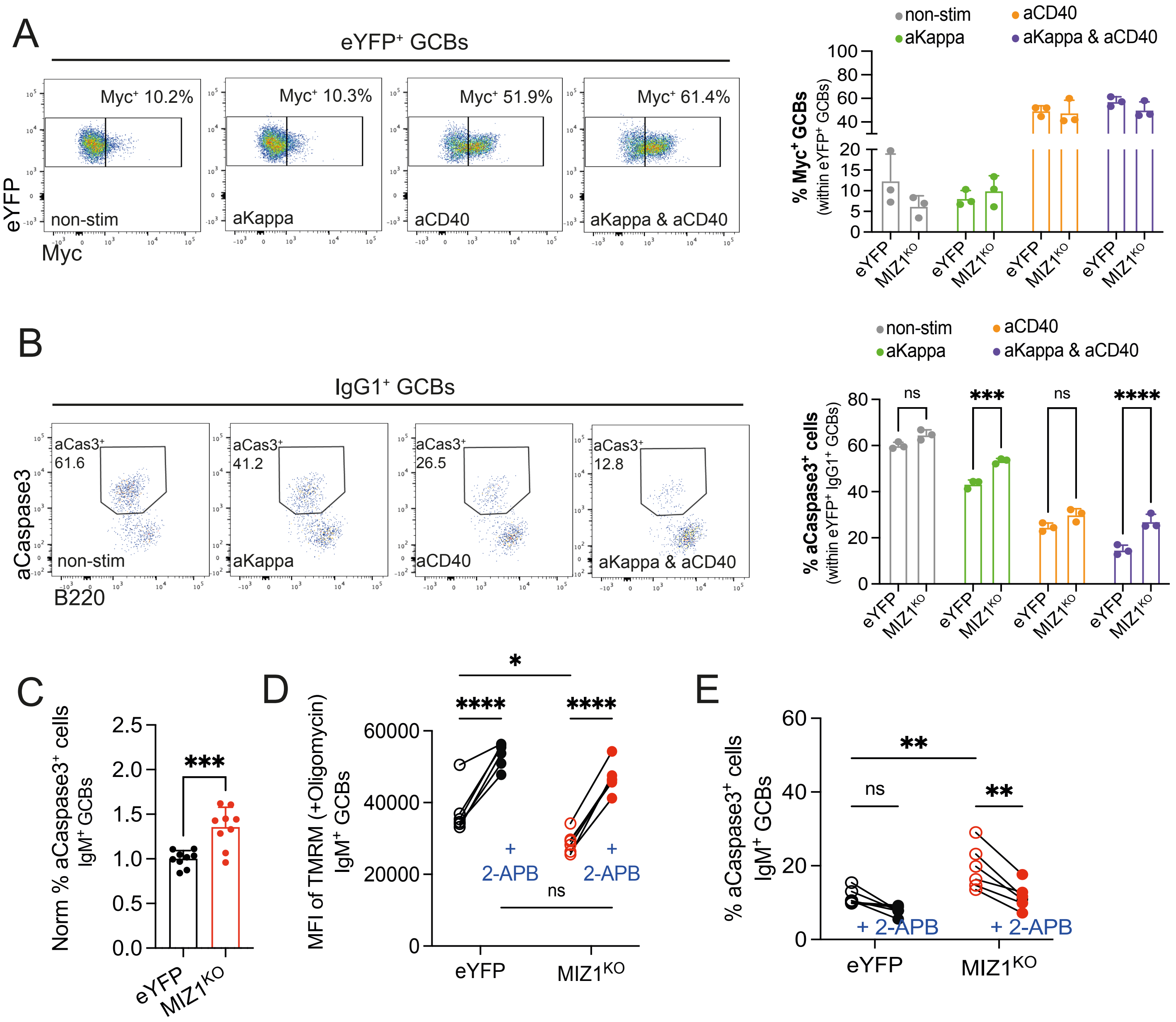

Zhang, et al.
